# A Review on Synthetic Fibers for Polymer Matrix Composites: Performance, Failure Modes and Applications

**DOI:** 10.3390/ma15144790

**Published:** 2022-07-08

**Authors:** Dipen Kumar Rajak, Pratiksha H. Wagh, Emanoil Linul

**Affiliations:** 1 Department of Mechanical Engineering, G. H. Raisoni Institute of Business Management, Jalgaon 425002, MH, India; 2 Department of Mechanical Engineering, G. H. Raisoni Institute of Engineering and Technology, Pune 412207, MH, India; waghpratiksha90@gmail.com; 3 Department of Mechanics and Strength of Materials, Politehnica University Timisoara, 300 222 Timisoara, Romania

**Keywords:** synthetic fibers, FRP composites, properties, failure modes, applications

## Abstract

In the last decade, synthetic fiber, as a reinforcing specialist, has been mainly used in polymer matrix composites (PMC’s) to provide lightweight materials with improved stiffness, modulus, and strength. The significant feature of PMC’s is their reinforcement. The main role of the reinforcement is to withstand the load applied to the composite. However, in order to fulfill its purpose, the reinforcements must meet some basic criteria such as: being compatible with the matrix, making chemical or adhesion bonds with the matrix, having properties superior to the matrix, presenting the optimal orientation in composite and, also, having a suitable shape. The current review reveals a detailed study of the current progress of synthetic fibers in a variety of reinforced composites. The main properties, failure modes, and applications of composites based on synthetic fibers are detailed both according to the mentioned criteria and according to their types (organic or inorganic fibers). In addition, the choice of classifications, applications, and properties of synthetic fibers is largely based on their physical and mechanical characteristics, as well as on the synthesis process. Finally, some future research directions and challenges are highlighted.

## 1. Introduction

The necessity for Synthetic Fibers (SFs) is advancing globally, as it is a highly significant form of material for fiber-reinforced composite structures. The growing demand for lightweight and unique composite materials increases the need for SFs due to their excellent characteristics [1,2,3]. There is strong competition between Synthetic Fibers and Natural Fibers (NFs) [4]. However, over the past 20 years, SFs such as ceramic fabrics, carbon fibers, glass fibers, basalt fibers, and polymeric fibers have received special attention [5,6]. Because SFs provide high strength and inertia, they are widely used in the production of advanced composite materials in aerospace and automotive environments [7].

On the other hand, due to the environmental sustainability, NFs are employed as a standby for SFs [8]. The flexibility and elastic properties of SFs make them stronger than NFs, also the water strain, chemical, and heat resistance capability of SFs are normally higher than that of NFs [9,10,11]. The shortage of NFs is satisfied by SFs, as the worldwide supply of NFs is insufficient [12]. In recent years, the SF business has grown very fast due to the growing demand for various applications [13,14,15]. The use of SFs in fiber-reinforced composites provides special characteristics compared to conventional high-density metals [16,17]. Some features include excellent tensile strength, electrical conductivity, extraordinary strength-to-weight ratio, and fatigue stability [18,19]. In the aerospace, defense, automotive, wind energy, and pipes manufacturing industries, there is a huge demand for composites based on glass, carbon, and aramid fibers [20].

The synthetic fiber has to pass through four processes at the time of manufacture, such as chemical, spinning, winding, and packaging. A chemical method is normally polymerization, which plans and consolidates the parts for the fiber. Polymerization is the development of macromolecules through the reiteration of essential units. At first, the different parts are solids and should be changed over to a fluid state to be expelled into fibers. The materials are synthetically changed over, disintegrated, or liquefied, transforming into a thick fluid. Further, the spinning cycle delivers the fiber by passing the thick fluid through a spinneret. A spinneret is a gadget with many openings of a predefined breadth. The fluid is constrained through the spinneret openings and comes out as string fluid fiber. The opening in the spinneret decides the measurement of the fiber, which is set by the application. The expulsion is dried to a ceaseless fiber. A winding cycle turns the fiber into yarn. The fiber falls upward from the spinneret and is trapped in a huge vacuum spout. The vacuum power makes pressure stay available, as it is twisted around a bobbin and packed for further use [21]. However, the most widely used process for obtaining fibers is electro-spinning. It is a manufacturing strategy involving electrostatically driven interaction that is used to make electro-spun fibers. The distance between these fibers varies regularly between many nanometers and a few micrometers. One of the main advantages of the electro-spinning process is its flexibility in handling to make filaments with different options for action and morphological constructions. With the development of nanotechnology, scientists are becoming more interested in focusing on the remarkable properties of materials on the nanoscale. Electro-spinning, an electrostatic fiber manufacturing method, has recently attracted more interest and attention due to its adaptability and potential for applications in various fields, being created or liquefied using a fixed electric field in a polymer assembly. Fibers created by this procedure emulate parts of the extracellular network very carefully in contrast to regular strategies. The twisted filaments in the submicron range created by this interaction offer various advantages such as a high surface area to volume ratio, adjustable porosity, and the ability to control the nanofiber structure in order to obtain the desired functions and properties [22].

Recently, SF has become the most demanding material on the market for fiber-reinforced composite. All types of composites reinforced with SFs are equally important for different grades of industrial applications [23]. Although the composite matrix is a constant part in the whole volume of the composite material, the discrete components in the volume of the matrix are reinforced parts or fillers, which largely decide the properties of the composite [24]. All composite polymeric materials can be restrictively grouped according to the components: the type of reinforcement and its direction, the type of matrix material, and the technique of manufacturing composites [25]. The important aspect related to SFs revealed that it significantly improves the load-carrying capacity with improved toughness, fatigue, and impact resistance, thus making it compatible with concrete for construction-related works [26,27].

Therefore, given the important and widespread distribution of SFs, an overview of the various SFs applicable to the composites sector is presented. In this paper, the SFs are divided into specific categories and for each type, the main characteristics and applications are presented in detail. The structure and arrangement of the fibers into composites together with their failure modes are also illustrated. Finally, some future research directions and challenges of using SFs are presented.

## 2. Types of Synthetic Fibers

**Figure 1:**
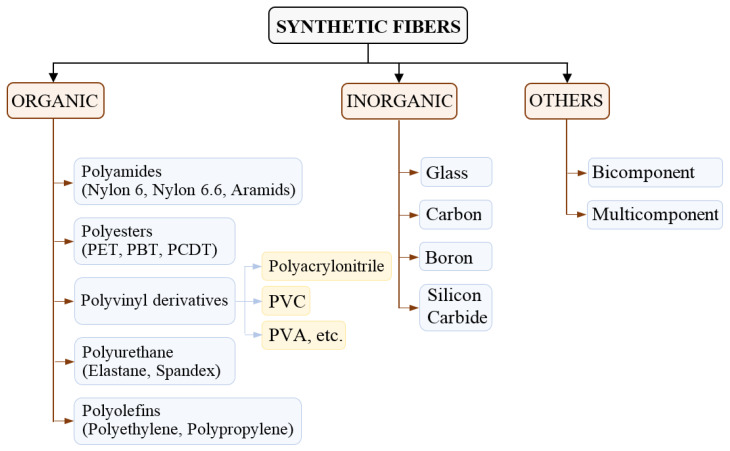
Classification of synthetic fibers based on organic, inorganic, and other fibers.

Synthetic Fibers are basically classified into three major categories: organic fibers, inorganic fibers, and others, which are further sub-classified according to their origin. The broad classification of SFs and their sub-classifications are illustrated in Figure 1.

Synthetic fibers are manufactured entirely in the laboratory from polymers that do not occur naturally, generally from petroleum by-products. Spinning, polymerization, and filament processing are the technique that can process synthetic fibers. Moreover, further spinning process is classified into three sub-categories such as melt spinning, wet spinning, and dry, to make filaments from synthetic polymers [21]. A viscous polymer is passed through a spinneret (a small nozzle-like plate with multiple small holes) to create a filament. Polymerization is a process where the material can be converted into high molecular weight complexes with various physical properties. Polymer is the name of a by-product spun from the reaction [28]. Numerous infinite fine filaments are formed in synthetic filament yarns. Continuous filaments are cut to short lengths to make staple fibers, which are then veiled and sold to the textile industry to produce yarn. The third form in which synthetic fibers are provided is yarn, which is also made from tow.

### 2.1. Organic Fibers

Organic fibers are obtained from a commodity that matures in the soil or is obtained from the skin of animals or is created by an insect. Abaca, camel hair, jute, hemp, coir, aramid, and silk are some common examples of organic fibers and there is a tremendous variation in the softness of substances made from these fibers. The fabulous fiber grows from the cocoon and the single extended strand is known as silk, which is obtained from the cocoons of a special caterpillar that serves on mulberry [29,30].

#### 2.1.1. Aramid/Kevlar Fibers

**Figure 2:**
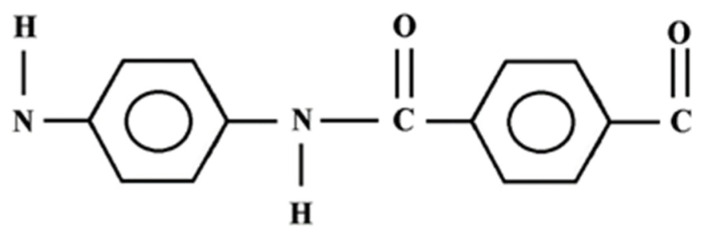
Primary structure of Aramid/Kevlar [33].

Aramid (AR) fiber is a kind of strong synthetic fiber, with outstanding properties, such as high-temperature stability, impact stability, low weight, and ensures outstanding energy absorption performance [31]. The structure of the Aramid fiber is the one shown in Figure 2. The structure of the aramid consists of several repeating connections between chains. These chains are connected with hydrogen bonds and offer ten times the tensile strength of steel for the same weight. AR fibers are twisted firmly to the point that it is beyond difficult to isolate them [32].

**Figure 3:**
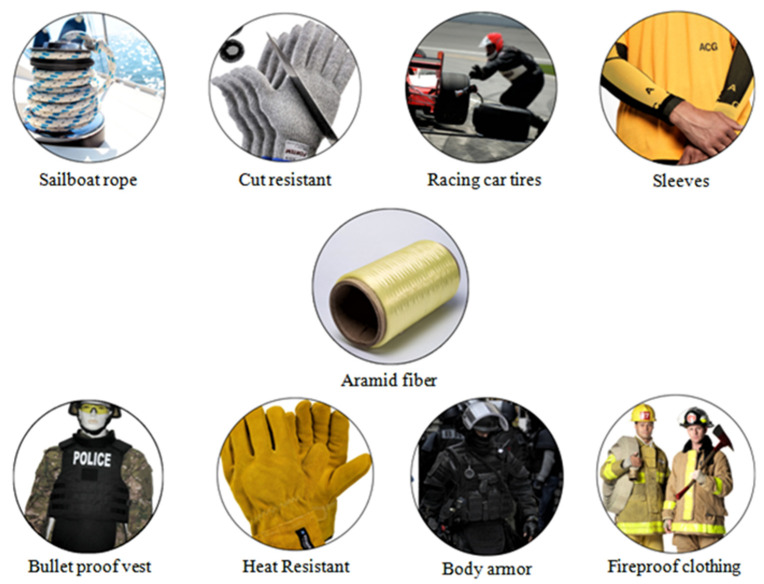
Branches of aramid fiber applications.

Aramid fibers are 45% lighter than glass fibers, twice as strong as E-glass, and ten times more efficient than aluminum [34]. The main use of aramid fiber is in the airplane and military industries, such as ballistic composites and ballistic-rated body armor fabric [35]. Also, other applications like bulletproof vests, boats, motorsports, protective gloves, and racing car body components are quite widespread [36,37,38]. Figure 3 shows the main applications of AR fiber [39].

Table 1 and Table 2 show the properties and global manufacturing of AR fibers. It was found that the sum of the engaging and opposite electric forces between atoms and hydrogen molecules and p-p stacking bonds has flourished the construction of Kevlar aramid nanofiber (KANF) based membranes for the separation and energy transformation of powerful bonds with the material capability. According to this information, several designs and approaches for manufacturing and practicing KANF-based membranes should begin [40].

In addition, the chopped AR fiber-reinforced rubber composite has been extensively used in structural applications due to its unique features. The hyperelastic characteristics of the rubber matrix are indeed a big challenge related to the extensive aspect ratio and the anisotropy of the AR fibers. Gao et al. [41] conducted a combined experimental-numerical study to predict the hyperelastic mechanical characteristics of the chopped AR fiber-reinforced rubber composite. The authors noted that, with the help of the digital image correlation, the mechanical deformation can be predicted efficiently, but the concentration of regional strain at the micron scale, depending on the macro image, is not accurately analyzed. 

#### 2.1.2. Polyethylene Fibers

**Figure 4:**
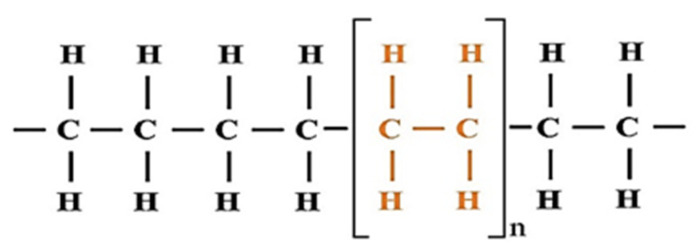
Chemical structure of polyethylene [46].

Polyethylene (PE) is made from the polymerization of ethylene and it is an adaptable and lightweight synthetic resin [44,45]. The chemical structure of PE is shown in Figure 4. PE is a thermoplastic polymer with a variable crystal structure and, contingent upon the kind, has a wide scope of uses.

**Figure 5:**
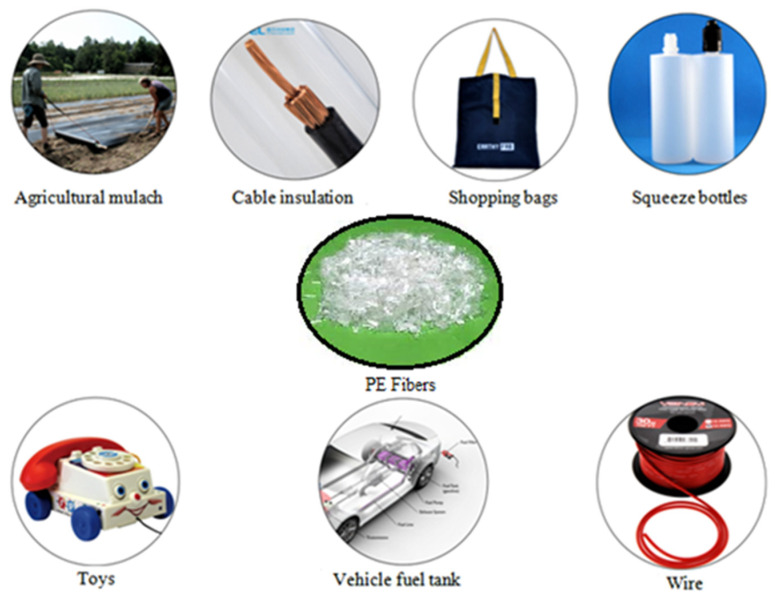
Various branches of polyethylene fiber application.

Polyethylene is the most widely used plastic in society, used in many applications, such as shopping bags, containers, oil spill cleaning, and vehicle fuel tanks [47]. The main properties of the PE compound are given in Table 3 and the applications in Figure 5.

Polyethylene fiber aggregates are low-density, linear, high-density, and ultra-molecular weight polyethylene (UMWPE) [48]. Low-density polyethylene (LDPE) is the primary grade of polyethylene with a strong, rigid, and crystalline organization. The wide range of polyethylene has high electrical insulation properties, higher thermal insulation, and great sliding properties, while their mechanical properties are generally moderate [49,50,51]. A crosslinking approach to the carbon chains of the plastic polymer has been discovered to create exceptional PE fibers that have a higher thermal obstruction than standard PE fibers. Contingent upon the level of cross-linking, the liquefying conduct of our PE fiber changes. While a slight cross-linking prompts a more viscous melt, unequivocally cross-linking filaments do not dissolve at all and show elastic-like, flexible property when heated. Due to the protected cycle, it can be easy to separately change the higher thermal resistance of the fiber and the higher consistency of the fiber melt, along these lines making an ideal reason for the utilization of PE fiber in a wide assortment of spaces of the variety of industry. With polymer dissolving points of up to 131 °C, PE fiber does not approach the thermal resistance of polypropylene or polyester filaments, yet they are all things considered appropriate for various applications. Also, some researchers are trying to produce new UMWPE composites with various reinforcements for the enhancement in the administration of properties, a pair of artificial intelligence methods were also been utilized for artificial neural networks and generative algorithms [52,53]. From these two methods, the most appropriate answers were achieved from the generative algorithm and artificial neural network principles were employed as the objective functions [54]. If composites like trans-1, 4-polyisoprene (TPI)/LDPE/carbon nanotubes (CNTs) were developed by a very simple natural blending technique, CNTs served as not only reinforced fillers but also nucleation promoter, which advances the crystallinity of the TPI and LDPE parts of the composites. Researchers conclude that the CNTs doses of 1 PHR (Parts per Hundred Rubber) could significantly improve the properties of the TPI/LDPE composites other than any CNTs doses [55,56,57,58].

#### 2.1.3. Aromatic Polyester Fibers

**Figure 6:**
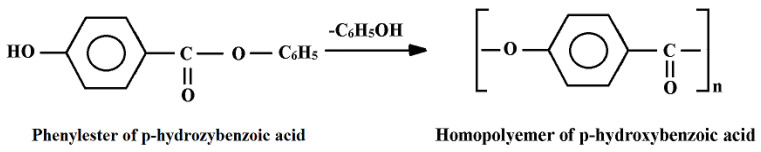
Aromatic polyester chemical structure [56].

Aromatic polyester fibers have noticed quite a few industrial uses because they have very high crystallinity, so they are difficult to process [59]. These types of polyesters fibers (polyethylene terephthalate, polybutylene terephthalate, polytrimethylene terephthalate and polyethylene naphthalate) have huge softening points, high-grade dielectric strength, superior mechanical characteristics, and excellent heat stability [60,61,62]. They are developed from aromatic dicarboxylic acids by polycondensation process, and some additives were added to improve the properties of the aromatic polyesters. PET (polyethylene terephthalate) is the most widely used polyester. Figure 6 shows the production of PET by self-ester exchange of the phenyl ester of p-hydroxybenzoic acid.

**Figure 7:**
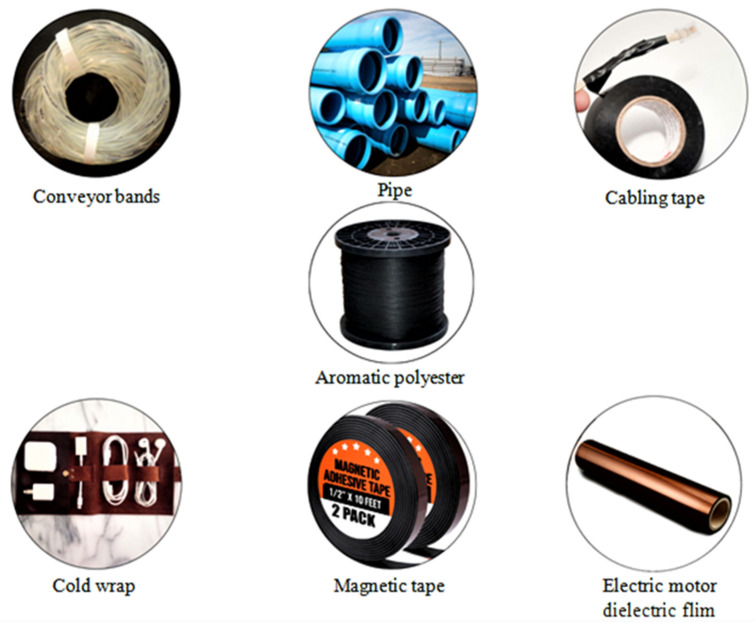
Application related to the aromatic polyester fiber in various sectors [63,64].

As shown in Figure 7, PET fibers are found in applications, such as conveyor bands, pipe, cabling tape, cord wrap, magnetic tape, and electric motor dielectric film [63,64]. These types of aromatic polyester fibers are created using a complex spinning process that require the recovery and reuse of strong polymer solvents [65]. The sub-atomic weight of the polymer is created in the acid arrangement, and when an objective sub-atomic weight is reached, then, at that point the arrangement is turned to shape continuous fibers. The use of solid acids presents difficulties in controlling and guaranteeing safety and harmlessness to the ecosystem rehearses for dissolvable caretaking.

It was observed that, comparing the mechanical properties of aromatic polyester fibers with other engineering materials, the polyester fibers have a tensile strength up to five times higher than aluminum [66,67]. These types of fibers are required where it is necessary to manufacture materials with less elongation and excellent strength. Also, the aromatic polyester fiber, in the thermosetting structure, is a superior/high-temperature polymer innovation, which is at the standard with the traditional epoxy-subordinate resin and elite design thermoplastics for the purpose of their probable applications. The aromatic matrix-based thermosetting nanocomposites show incredibly upgraded actual properties empowered by a science-supported vigorous interfacial covalent coupling instrument created during the in situ polymerization response with different nanofiller molecule setups [68].

In addition, efforts are being made to develop high-performance fire-resistant epoxy resins with phosphorus-containing semi-aromatic polyester, used as multi-functional epoxy filler, with the collective enhancement of fire-resistance and mechanical properties for various possible applications of production [69,70]. Table 4 presents the main properties of the PET compound.

The most commercially polyester-based fiber available today is liquid crystal aromatic polyester (LCP) fiber. The physical properties of LCP fibers equal and sometimes exceed those of aromatic polyamides (aramids). The main advantages of LCP over other competitive fibers are abrasion durability, dimensional/chemical stability, and flex–fatigue resistance, for example, in repeated wash industrial cycles.

#### 2.1.4. Nylon Fibers

Numerous apparel and consumer goods are typically made from the family of synthetic polymers known as nylon. Unlike other organic or semi-synthetic fibers, nylon fibers are synthetic, which means they do not have an organic base. Nylon fibers are far more robust, elastic, and long-lasting than polyester fibers. The fibers can be dyed in a wide range of colors and are very strong, abrasion-resistant, easy to wash, and strong. The filament yarns provide a smooth, supple, lightweight fabric with good resilience. Nylon is used in both the textile and home goods industries. However, because of its lower wrinkle resistance and higher price, polyester has replaced cotton in many garment products. Monomers, which are extended chains of carbon-based molecules, are the building blocks of the polymer nylon fabric. Although nylon is available in many different forms, the majority of them are made from polyamide monomers that are derived from petroleum, or crude oil. Adipic acid and diamine acid react to create the polymer known as nylon. This polymer, also known as PA 6, 6, was the first kind to be utilized for nylon fabric. The substance PA 6, 6 is an illustration of a nylon salt since it transforms from a crystalline state to a molten state when heated [29]. Below some important types of nylon composite are shared [30] in Table 5.

### 2.2. Inorganic Fibers

Nowadays inorganic fibers are becoming more and more widespread. The most common fibers manufactured from inorganic materials are glass, carbon, boron, silica carbide, alumina, potassium titanate, and ceramics [72,73,74]. Inorganic fibers can be divided into three main categories, such as amorphous, polycrystalline, and monocrystalline fibers. Due to the lack of grain boundary, amorphous fibers exhibit high strength and moderate modulus of elasticity [75]. Polycrystalline fibers are made of small crystals, so they have excellent heat stability. Monocrystalline fibers are like very fine fibers, showing extremely high strength properties [76].

#### 2.2.1. Glass Fibers

**Figure 8:**
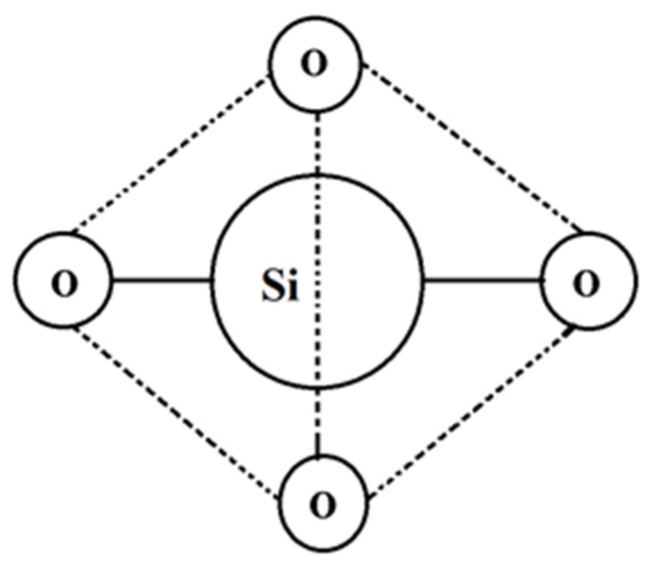
Chemical structure of glass fiber [82].

Glass fibers (GFs) are fabricated by melting silica sand, limestone, boric acid, and some other components at a very high temperature of about 1200 °C above [77]. Also, in the obtained composition some oxides of several metals are added. Glass fiber is extremely fine, lightweight, high strength, and also very durable material [78]. Compared to carbon fiber (CF), GFs have lower strength but are less expensive than the CFs [79]. Glass fiber can be easily formed using casting methods, and when related to metals, GFs are very profitable due to their strength and weight characteristics [80,81]. Figure 8 shows the chemical structure of GF, a non-crystalline material with a short-range network structure. As similar, it does not have a definite microstructure and therefore the mechanical characteristics, that area unit considerably determined by composition and surface finish, are identical [82].

**Figure 9:**
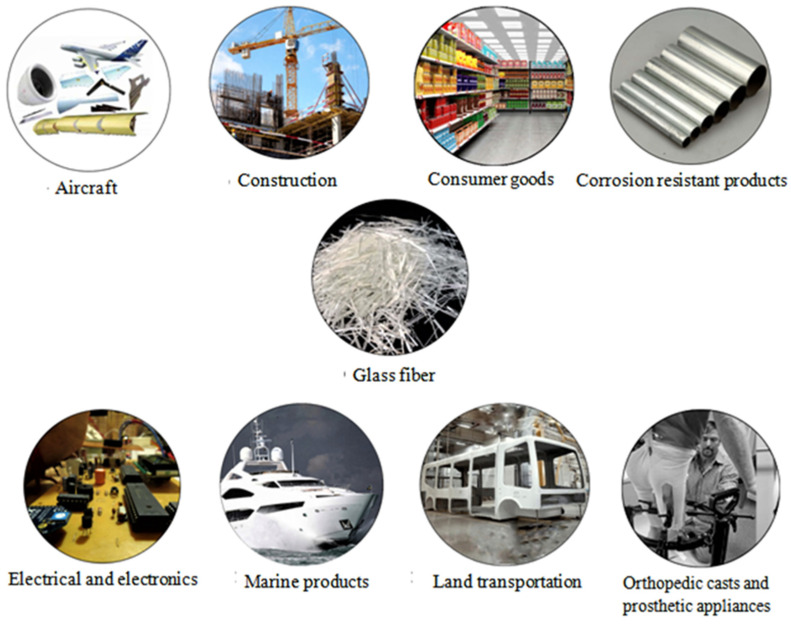
Glass fiber applications in various sectors.

Due to its high efficiency at extended temperatures, the GF reinforced epoxy composites are frequently used in high voltage insulation applications. The addition of nano and micro fillers in epoxy improves the dynamic mechanical characteristics of GF reinforced epoxy composites [83,84]. In addition, for reducing the weight and enhancing the hardness and strength of the automobile parts, an eco-friendly polymer composite can be used [85,86]. In this case, the epoxy resin represents the matrix of the composite, and the glass and palm fibers strengthened reinforcement [87]. It has been reported that by increasing the phase ratio of the fiber particles, the tensile strength of the composite is significantly improved [88]. Moreover, one of the more important properties property in composite design is the inter-laminar shear strength of composites, which is very essential for the application of laminated composites. Therefore, to enhance the interlaminar shear strength of composites, some oxides such as graphene transformed short GFs have been introduced between the glass fiber and epoxy matrix [89]. The main applications of GFs are shown in Figure 9.

**Figure 10:**
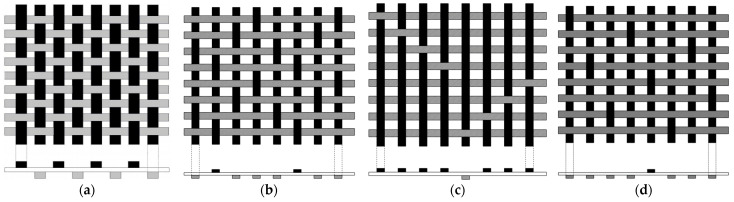
Various weave forms of GF (**a**) Plain weave (**b**) 4-harness satin weave—120GF (**c**) 8-harness satin weave—1581GF (**d**) 8-harness satin weave—181GF [92].

To obtain the desired properties, GFs are organized in different ways [90]. Figure 10 shows some types of fiber arrangements. The strength of the GF composites depends on the type of weave. Wherever the uniformity is preferred for strengthening, the square division’s even number per inch of edges in the thread as tools, plain weave holding is operated [91].

The 8-harness satin weaves are used when the lamination strength from all the sides, as well as even exterior and beautiful presentation, is wanted. In this 8-harness satin weave, the filling fiber passes over seven twist fibers and next below one thread fiber. The more flexible than the plain weave is the 4-harness satin weaves, here the filling fiber passes over three thread fibers and then below one thread fiber [92]. Glass fiber is usually employed in the structural application of aircraft like fairings, radomes, wingtips, and helicopter rotor blades. To withstand a large current flow E-GFs are used because they are made of borosilicate glass. If higher strength E-glass is required, then S-glass is used. There are five grades of GF, such as general-purpose, quartz, protective, hollow, conducting/semi-conducting GFs and the main types of GFs are presented in Table 6 [81].

▪ A-glass is the most common type of silica glass and is also called soda-lime-silica glass. This type of GF is sensitive to temperature variations, due to the high coefficient of thermal expansion produced by high thermal stresses that can cause yielding.
▪ E-glass is a fungal or bacteria-free fiber that can withstand the main chemical causes, and endures dimensionally steady, even under extreme fluctuations of moisture and heat.
▪ C-glass is practiced for making glass screens or shades lacking corrosion-resistant properties.
▪ AR-glass is produced mainly for coating standard cement weapons. It can resist the alkaline aggregates produced throughout solidification as it contains high zirconium oxide.
▪ S-2-glass is normally used for polymer matrix composites that necessitate improved mechanical characteristics. S-glass fiber is used for high-performance applications.
▪ D-glass is a low dielectric constant glass produced with borosilicate and used in electrical applications.
▪ R-glass is a reinforcement glass made of calcium aluminosilicate used where higher strength and acid corrosion protection are required.

#### 2.2.2. Carbon Fibers

Carbon fiber (CF) is made of thin, strong crystalline carbon filaments, and aims to strengthen the developed composite materials [93,94,95]. The diameter of the CF can reach up to 5 microns (sometimes below), increasing its strength when twisted together like yarn. CF is sometimes identified as a graphite fiber. CF has many advantages including high stiffness, high tensile strength, low weight, high chemical resistance, high-temperature tolerance, and low thermal expansion [96,97,98]. These facilities have made CF very widespread in space, architecture, military, and motorsports. CF is twice as stiff as steel and five times stronger. All these features make this material ideal for the manufacture of various parts [99,100]. Despite this, they are comparatively costly when correlated with comparable fibers such as glass or plastic fibers [101]. CF is produced from a mixture of chemical and mechanical methods. In the chemical method, light heating of the polymer to 600 °C, which discharges hydrogen gas and adjacent polymer chain detonators. By additional heating to 1300 °C, different chains can fuse each other because they excrete more hydrogen and nitrogen; the remaining pure sheet is of carbon atoms.

**Figure 11:**
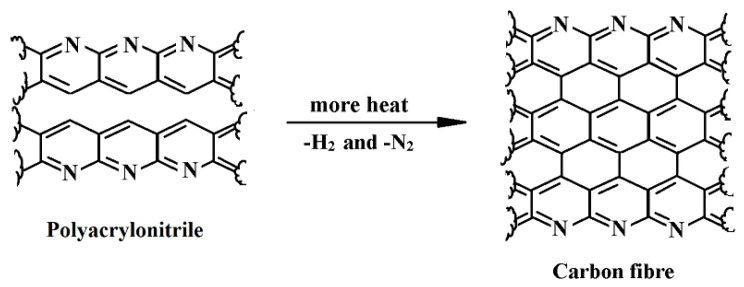
Chemical structure of carbon fiber [102].

The high temperature and pressure involved in this method straighten the tiny crystals that the carbon sheet makes all adjacent to the corresponding axis. The chemical structure of CF is shown in Figure 11 [102].

**Figure 12:**
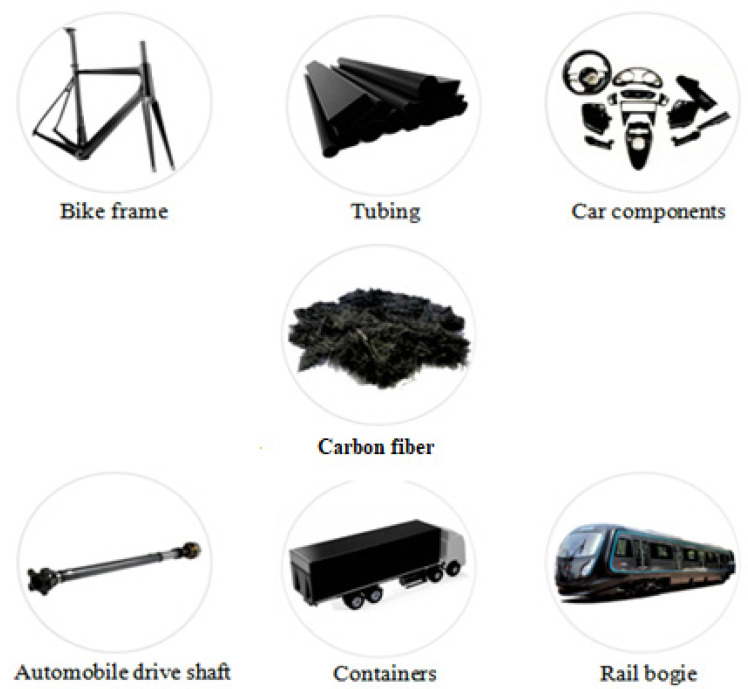
Application of carbon fiber in various sectors [106].

Carbon fiber reinforced polymer (CFRP) may have poor electrical conductivity, which influences some safety issues when exposed to lightning in aerospace applications [103]. Therefore, the remarkable layer of transformed silver carbon nanotube was promoted through the electrophoretic deposition technique for the preservation of CFRP structures and parts [104]. Carbon fibers have gradually replaced many traditional materials and have confirmed that by adding CF, the properties can be improved because CF rods are used rather than steel bars [105]. All these characteristics have led to an increase in the applicability of the carbon fibers, Figure 12 [106].

**Figure 13:**
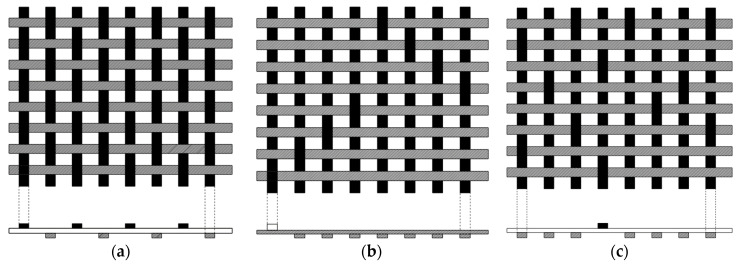
Weaves of carbon fiber (**a**) Plain weave—3K-70-P carbon (**b**) 8-harness satin weave—3K-135-8H carbon (**c**) 5-harness satin weave—1K-50-5H carbon. Reproduced from [107].

Depending on the CF weaves, the strength of the fiber can be determined. For this, the different weaves, such as plain weave, eight-harness satin weave, give-harness satin weave, are shown in Figure 13. Due to poor layering, the plain weaves are used for simple shapes or structures, while eight-harness satin weave exhibits improved fiber flexible property.

The GFs are divided into ultra-high modulus (UHM) (E > 450 GPa), high modulus (HM) (350 GPa > E > 450 GPa), intermediate modulus (IM) (200 GPa > E > 350 GPa), high tensile strength (HTS)-low modulus (LM) (σ > 3 GPa and E < 100 GPa), ultra-high tensile strength (UHTS) (σ > 4.5 GPa) [108,109,110,111], includes the main physical characteristics of GFs are presented in Table 7 [108,112,113] which is depending on the mechanical behaviors.

In addition, according to the manufacturing method, the GFs are divided into [114]:

▪ PAN type CF, generated from the carbonization procedure of polyacrylonitrile at a temperature of 1200 °C for several minutes. These fibers have an HTS and HM, being widely used in the aerospace and sports industries [115].
▪ Pitch type CF, generated from the carbonization procedure of oil/coal in a nitrogen environment at a temperature of about 1200 °C. Pitch CF has various characteristics from LM to UHM. Due to this aspect, the fibers are used in stiff and thermally conductive elements [116].

#### 2.2.3. Boron Fibers

Boron fiber (BF) is an amorphous elemental boron outcome widely adopted in aerospace utilization due to its high strength and lightweight characteristics [117,118,119]. It is fabricated by chemical vapor deposition (CVD) of boron at a temperature of 1000 °C on a substrate and obtained fiber is called boron fiber. Due to the internal residual stresses in the BF, a significant impact on the fiber mechanical properties takes place at the time of CVD as the boron trichloride (BCl3) is mixed with hydrogen and boron, is deposited according to the reaction given by Equation (1).

2BCl3(g) + 3H2(g) → 2B(s) + 6HCl(g)(1)

**Figure 14:**
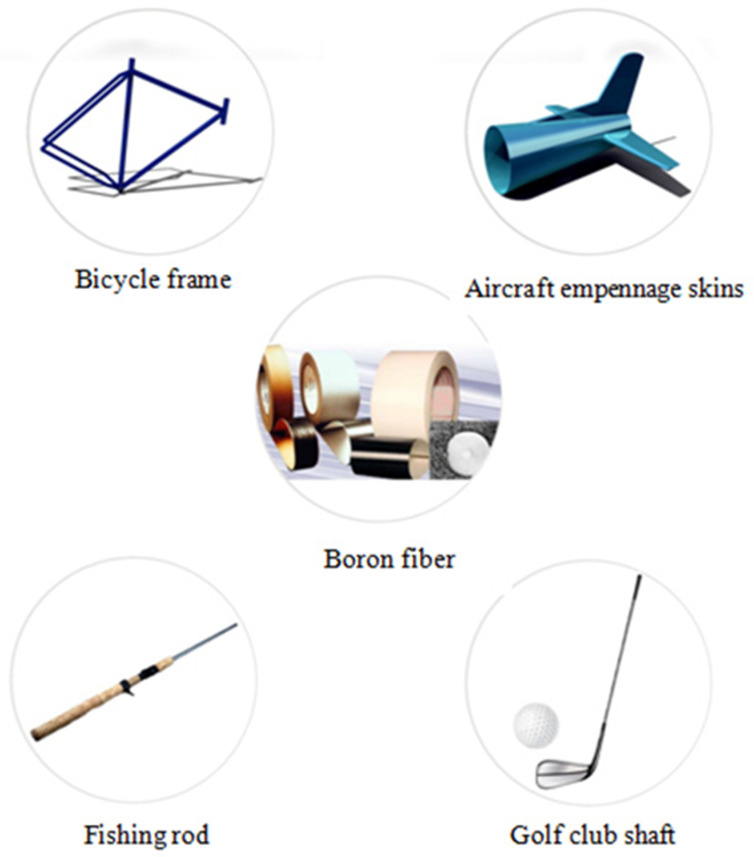
Main applications related to boron fiber.

In the transverse region of boron fiber, the radial crack would usually be observed due to the internal stresses. The structure of the BF depends mainly on the three deposition provisions, such as temperature, gas composition, and gas dynamics [120,121,122]. The disadvantage of boron fiber is the high cost compared to other fibers, being used mainly in several U.S. military aircraft, especially F-14 and F-15, and in the space shuttle [123,124,125]. In addition, it can be used for the renovation of the metal constructions [126]. The main applications of BFs are shown in Figure 14.

To improve fatigue crack initiation life (FCIL) and to reduce stress levels in the joint layers, it is necessary to increase the Young’s modulus of each layer. In this regard, Chang et al. [127] performed an experimental investigation of the fatigue crack behavior of two different hybrid boron/glass/aluminum fiber/metal laminates (FML). They observed that the inclusion of BFs has improved Young’s modulus of composite layers in FMLs. In addition, their experimental results showed that FCIL for both FML composites was superior to monolithic aluminum alloys under the same loading conditions. Next, an analytical approach was suggested by the authors for the calculation of FCIL of FML based on both the small-crack theory and the classical laminate theory. Table 8 summarizes the main properties of the BFs [128].

#### 2.2.4. Silica Carbide Fibers

**Figure 15:**
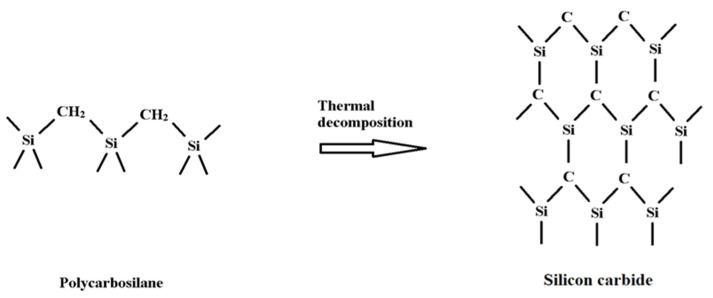
Chemical structure of silicon carbide fiber [132].

Silicon carbide (SiC) fibers are available in both alpha- and beta-SiC combinations. They are at the same time very fine flexible continuous fibers made from multiple woven fabrics [129,130,131]. The chemical structure of SiC fiber is given in Figure 15 [132].

**Figure 16:**
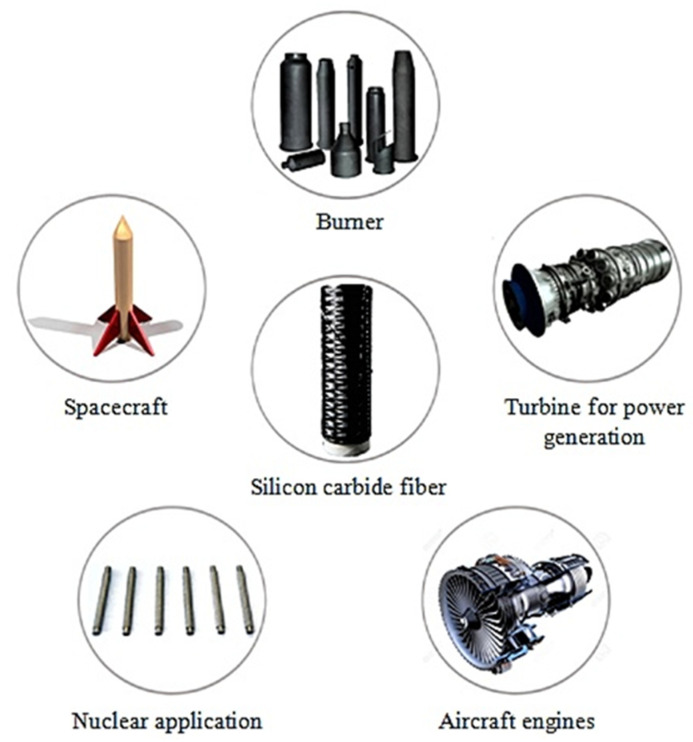
Image showing various applications of silicon carbide.

Compared to organic and some ceramic fibers, SiC fibers have unique characteristics such as high tensile strength, high stiffness, high modulus, high chemical resistance, low weight, low thermal expansion, and high-temperature tolerance [133,134,135]. SiC fibers have a considerably better durability compared to other fibers like carbon, glass, alumina, and alumina silicate [136]. These fibers do not react with normal matrix alloys, as the extraordinary fiber intensity is sustained up to 1200 °C in an inert gas environment [137]. Figure 16 shows the main applications of the SiC fibers.

Silicon carbide fibers are fabricated in many ways, such as chemical vapor deposition (CVD), laser-driven CVD (LCVD), and the Yajima process. For high molecular weight compounds, this process may vary depending on the fiber diameter [138]. In the CVD process, the SiC fibers are relatively larger in diameter (between 80 to 140 microns), compared to the corresponding LCVD (20 to 80 microns) and Yajima (20 microns) processes. Also, one process known as binder jetting additive manufacturing supported by polymer infiltration and pyrolysis was employed to manufacture the SiC fiber-reinforced composites to improve the properties like fracture toughness, har, dness and flexural strength.

To improve the oxidation properties of carbon fiber, SiC is coated on the carbon surface to be used for solar-cell ingot-growing containers. Currently, SiC fiber has a small market due to high cost, but can exhibit robust behavior under high temperature and oxidative environment conditions [139,140,141]. The main properties of SiC fibers are presented in Table 9 [142].

## 3. Failure Modes in FRP Composites

**Figure 17:**
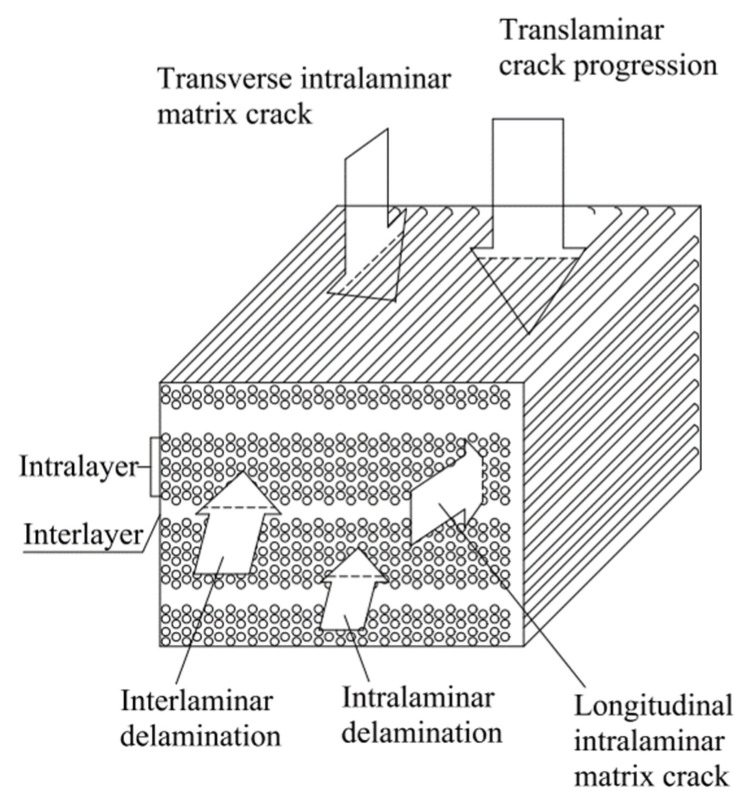
Delamination and cracks directions [143].

The main causes underlying the failure in FRP composites are represented by delamination, debonding, micro-cracking of the matrix, and breaking of fibers. As presented in Figure 17, the fracture modes in FRP composites can be divided into three basic fracture types: inter-laminar, intra-laminar, and trans-laminar [143].

**Figure 18:**
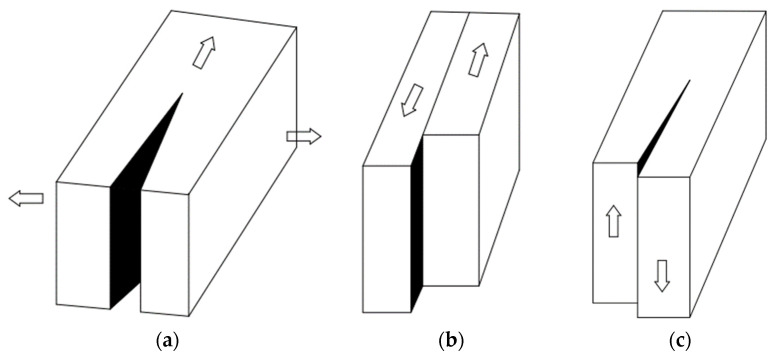
Crack opening modes: Mode I or opening/tensile-mode cracks (**a**), Mode II or sliding/in-plane shear (**b**), and Mode III or tearing/out-of-plane shear (**c**) [147].

The delamination failure modes, as shown in Figure 18, are normally categorized into mode I, mode II, mode III, and mixed-mode (I + II, I + III, and II + III) delamination modes, according to the principal stresses appearing on the interface [144,145,146]. If considering the failure on the micro-scale inter-laminar and intra-laminar fracture types can be thus characterized.

In both inter-laminar and intra-laminar failure modes, fracture occurs on a plane parallel to that of the fiber reinforcement. The fracture of each type can occur under pure mode I (opening/tensile-mode cracks), mode II (sliding/in-plane shear), and mode III (tearing/out-of-plane shear), or under a mixed mode (I + II, I + III, II + III) [148,149]. Trans-laminar fractures are those located transverse to the laminated plane in which conditions of fiber fractures are created [150]. Manshadi et al. [151] had correlated the experimental results with computations of a semi-empirical basis by the plotting of the total critical strain energy release rate, and very good results were found i.e., total critical strain energy release rate increases as a function of mode II/total critical strain energy release rate with semi-empirical criterion exponents applied to delamination initiation and growth (mC) = 2.6, whereas total fracture resistance versus mode II/total critical strain energy release rate modal ratio is linear with semi-empirical criterion exponents applied to delamination initiation and growth (mR) = 1.

### 3.1. Delamination

**Figure 19:**
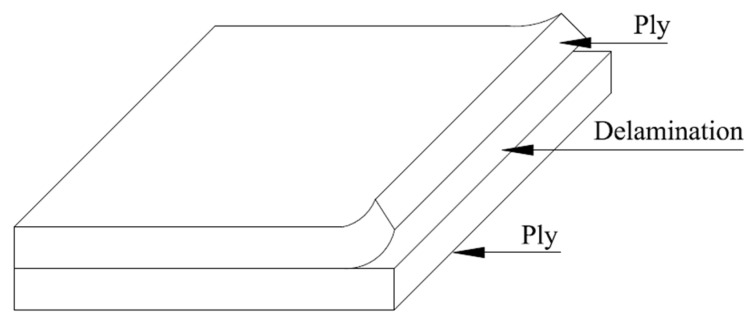
Separation of adjacent layers due to weakening of interface layer between them.

Delamination is a mode of failure in which material fractures into layers, as shown in Figure 19. It is a critical failure mode in composite structures, not significant because it will prompt the structure to fracture into two or more pieces, but because it can discredit the laminate to such a degree that it fits ineffective in service [152,153]. The interfacial detachment produced by the delamination may begin with too early buckling of the laminate, extreme vibration, the interference of moisture, stiffness degeneration, and lack of fatigue life. Delamination may be advanced during in-service conditions. It may transpire from low-velocity impact, from peculiarities in the structural load pathway that may cause great out-of-plane stress [154,155].

Analyzing mechanical loads, the moisture and temperature may also produce inter-laminar stresses in a laminate. It is due to the effects of the residual thermal stresses generated by cooling from treating temperatures and residual stresses produced by the consumption of moisture. The delamination may lead to the redistribution of stresses, which ultimately improves total failure. The strain energy released by the propagation of a delamination of length a to a + Δa is given by Equation (2), as follows [156]:

(2) $$W=\frac{1}{2}\int_{a}^{a+\;\Delta a}\;\int_{-\;\Delta a/2}^{\Delta a/2}\sigma \left(x,y\right)\delta \left(x-\Delta a,y\right)\mathrm{dx}\;\mathrm{dy}$$

where δ(x − Δa)—crack opening displacement, σ(x,y)—stress at the crack front required to close the delaminated area.

Benzeggagh and Kenane [157] evaluated the delamination growth in a unidirectional glass/epoxy composite under mode I, II and mixed mode I/II static loading configuration. The authors expressed the properties in terms of the total fracture resistance and the total critical strain energy release rate. Moreover, the experimental data were correlated with computations of a semi-empirical criterion. Furthermore, Berthelot [158] reviewed the delamination process in cross-ply FRP laminates under static and fatigue loading. In order to develop different models for evaluating the stress distribution in the damaged FRP laminates, the author used various experimental data. Berthelot [158] models, associated with the statistical description of strength or energy, allow describing the development of transverse cracks in monotonic or fatigue loading.

### 3.2. Fiber Pull-Out and Debonding

**Figure 20:**
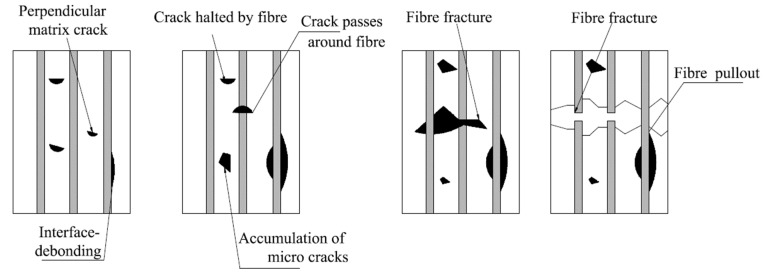
Fiber pull-out and debonding.

The fiber pull-out is one of the failure mechanisms that can occur in FRP composite materials. Weak bonding is the main cause of fiber pull-out and delamination. The cracks on the fibers are not injured at the leading distance but in the high-stress zone near the tip, they are broken and quickly behind the crack tip fibers pull out of the matrix as shown in Figure 20 [159].

In the case of some FRP composites, before they collapse, the stress near the crack tip may cause a debonding of the fibers from the matrix material. On the other hand, if the fibers are brittle and well bonded to a ductile matrix, the fibers tend to fasten forward of the crack tip, moving bridges of matrix that pet down and shatter in a perfectly ductile manner.

In addition to mentioned local failure mechanisms, on entering the interface of the two laminate in a FRP laminated composite, a crack can split and propagate along with the interface, thus offering the delamination crack [159,160].

Work debonding is calculated using Equation (3) [161]:

(3) $$W_{d}=\frac{\pi d^{2}\sigma_{f}^{2}l_{d}}{24E_{f}}$$

where *d*—fiber diameter, $\sigma_{f}^{2}$—failure strength of the fiber, $l_{d}$—length of the debonded zone, $E_{f}$—fiber modulus.

### 3.3. Microcracking

**Figure 21:**
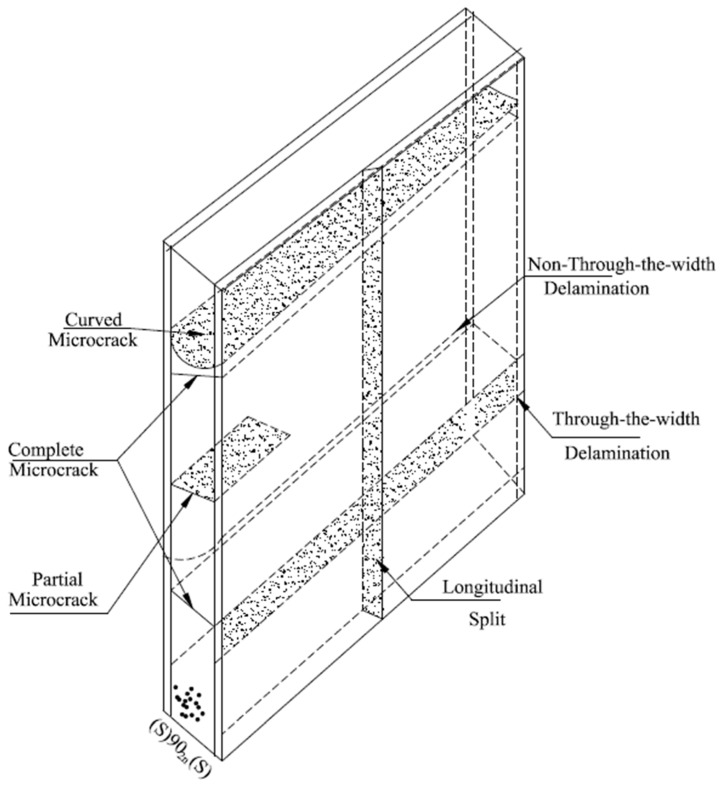
Microcracking types.

Microcracking is the very small cracks in the composites that are not visible to the naked eye. The primary form of damage in laminates is often matrix microcracking. They are intra-laminar fractures that traverse the width of the layer and move laterally to the fibers. The common noticeable microcracking is cracking in the 90° plies through axial loading in the 0° direction as shown in Figure 21 [162]. These microcracks are transverse to the loading direction and are often referred to as transverse cracks. Microcracks may be recognized during fatigue loading, tensile loading, thermo-cycling and during changes in temperature. Microcracks can develop in several plies but mainly they are exposed in the implies off-axis to the loading axis.

The quick effect of the microcracks is to generate degeneration in the thermo-mechanical characteristics of the laminate including variations in all practical modules. The extra harmful effect of the microcracks is that they create other forms of damage such as induction of delamination, fiber cracking, or afford pathways for the entrance of corrosive liquids. Such damage modes may consequently begin to laminate failure. The primary, microcrack produces surprisingly few differences in the thermo-mechanical characteristics of the laminate. Further loading normally starts as extra microcracks and supplementary microcracks and continues degeneration in the thermo-mechanical characteristics. The changes in the temperature influence residual stress within the plies and therefore can begin micro-cracking [162].

## 4. Fiber/Matrix Interface and Surface Treatment

### 4.1. Fiber/Matrix Interface

**Figure 22:**
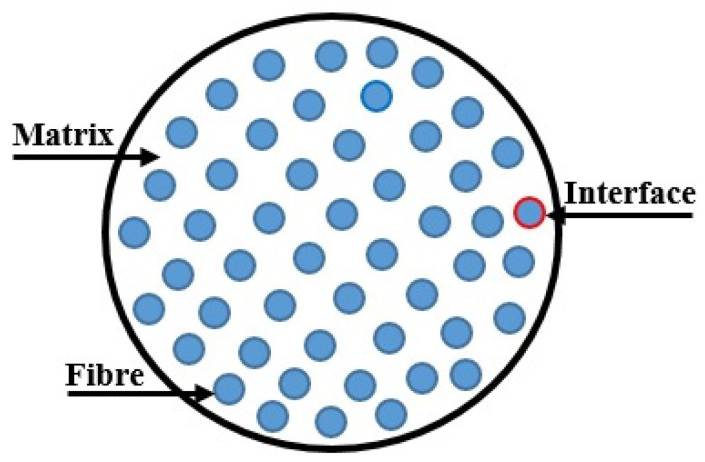
Fiber/matrix interface.

A line that divides fiber/matrix in a physically distinct and separable phase is termed an interface as shown in Figure 22. The fiber/matrix interface represents a very significant performance in predicting the macroscopic mechanical properties of the composites. While analyzing the interaction between fiber/matrix in the composite, 2-dimensional interphases are considered. At the time of discovering the properties of the composites, fiber/matrix properties such as the debonding strength and sliding resistance perform a dominant role. The commonly employed technique for measuring fiber/matrix properties is fiber push-out testing [163].

Following are the technique for measuring fiber/matrix properties in the composites:

- Direct methods: Single-fiber pullout tests, Multiple-fiber pullout tests, Fiber fragmentation tests, and Micro-indentation push-in tests.
- Indirect method: Short-beam shear strength test, Inter-laminar shear strength tests.

### 4.2. Surface Treatment

The surface treatment is usually performed in the composite material to reduce the weak boundary layers, developed wetting of low-energy surfaces, and chemical modification. The surface treatment intends to modify the morphology of a thin surface layer without altering the bulk characteristics.

The following are the most significant surface modification techniques used for better mechanical performance [163]:

▪ Chemical modification through treatment, CAs, and functionalization;
▪ Chemical modification through etching;
▪ Chemical modification through grafting;
▪ Physical modification through surface tension and energy compatibilization;
▪ Radio-frequency (RF) sputtering, chemical vapor deposition, physical vapor deposition, and plasma-assisted CVD;
▪ Cold spray for low-temperature polymers;
▪ Electrolytic, electro-less, and dip coatings;
▪ Physicochemical techniques as a combination of these techniques;
▪ Stem cell culture, cloning, and growth on unmodified or modified substrates;
▪ Laser, electron, plasma, infrared, and x-ray irradiation techniques to modify surfaces;
▪ Ultrasound, RF, and microwave sonication;
▪ Surface static charge and conductivity modifications;
▪ Surface roughness, texture, and topography modifications for mechanical adhesion;
▪ Neutron chemical transmutation doping of the reinforcement surface;
▪ Diffusion processes, surface-selective hardening, and softening techniques;
▪ Transverse fibrillation for superior bonding, phase transition modifications, and skinning and cladding;
▪ Preferred polymorphic and allotropic transformations that contribute to strengthening;
▪ Weaving, stitching, knitting, and braiding to improve transverse flow and provide more surface area, wettability, and percolation of the matrix;
▪ Coefficient of thermal expansion matching with the matrix for accommodative interfacial behavior;
▪ Sizing, thermal, and water boiling treatment;
▪ Vacuum and hot vacuum degassing to remove contamination; and
▪ Thermo-oxidative adhesive coatings to improve interfacial thermal stability.

## 5. Conclusions and Future Directions

The properties of SFs depend on the type of used fiber and its manufacturing process. Because, compared to conventional metals, SFs have superior mechanical, tribological, thermal, and chemical durability, their use for various purposes has been continuously increasing worldwide. Depending upon the chemical composition, SFs are categorized into organic, inorganic fibers, and others. Since ancient times organic fibers are found in our daily applications. On the other hand, compared to organic fibers, inorganic fibers are recently developed and have been shown to have remarkable properties, thus having a high potential for future research.

The main advantages of SFs are long-lasting, stretchable, waterproofing, moisture resistance, strain and wear resistance; while they also have many disadvantages such as being flammable, not being suitable for hot washing, cause for microplastic pollution, having poor insulation capacity, melting easily, being prone to heat damage, and not being eco-friendly. For the SFs to be used to their maximum potential they must be compatible with the matrix, to have physical and mechanical properties superior to the matrix, to present the optimal orientation in composite, and to make chemical or adhesion bonds with the matrix.

The biggest challenge facing manufacturers in terms of SF is its non-biodegradability and lower melting point. Due to such safety and environmental concern, SFs are lagging behind in the market. There is a great opportunity to focus on the research of SFs for the development of more technologies to provide better technical and safety features. Moreover, important attention should be paid to the integration of useful changes in SFs with less or no harmful influence on the environment some important attention has to be made. In response to these challenges, studies should be focused on SF-reinforced bioplastics, plastic nanocomposites, self-healing polymers, plastic electronics, smart and reactive polymers. Ongoing cross-disciplinary research will improve the biodegradation and biorecycling options of SFs.

## Figures and Tables

**Table 1:** Properties of Aramid/Kevlar composites [37,38,42].

Material	Matrix	Fiber Weight Fraction	Laminate Specific Gravity	Tensile Strength (MPa)	Tensile Modulus (MPa)	Specific Tensile Modulus (MPa)	Specific Tensile Strength (MPa)	Compressive Strength (MPa)	Compressive Modulus (MPa)
AR K49, Woven	Polyester	0.44	1.31	430	25,994	19,857	329	115	16,272
AR K49, Woven	Epoxy	0.55	1.31	450	29,993	22,890	344	172	-
Kevlar 49 (1350)	Polyester	0.42	1.293	372	19,306	-	-	115	17,927
Kevlar 49 (1350S)	Polyester	0.42	1.3	384	23,925	-	-	114	19,306
AR 900S	Polyester	0.48	1.294	443	26,614	-	-	103	19,306

**Table 2:** Global manufacturing of AR fibers [43].

Fiber Product	Base Polymer	Fiber Producer	Application
***p-AR fibers***			
Kevlar	Poly(p-phenylene terephthalamide)	DuPont Co.	Goods offered to give multi-threat security
Twaron	Poly(p-phenylene terephthalamide)	Akzo Nobel	PVB Prepreg
SVM	Poly[5-amino-2-(p-aminophenyl) benzimidazole terephthalamide]	Russia	Bulletproof vest and helmets
***m-AR fibers***			
Nomex	Poly(m-phenylene isophthalamide)	DuPont Co.	Flame barrier for aircraft insulation
Teijinconex	Poly(m-phenylene isophthalamide)	Teijin Ltd.	Hoses, filters, and copy cleaners.
Fenilin	Poly(m-phenylene isophthalamide)	Russia	Fire resistance application
***AR copolymer fibers***			
Technora	Copoly(1,4-phenylene/3,4’-diphenylether terephthalamide)	Teijin Ltd.	Radiation shielding
Armos	Copoly[p-phenylene/5-amino- 2—(p-aminophenyl)benzimidazole terephthalamide]	Russia	Open fire resistance
Trevar	Aramid copolymer	Hoechst AG	Aerospace and military

**Table 3:** Properties of polyethylene compound [56,57,58].

Properties	HDPE	LDPE	LLDPE
Melting point (°C)	120 to 140	105 to 115	115 to 135
Density (g/cm^3^)	0.941 to 0.965	0.910 to 0.925	0.91 to 0.94
Continuous temperature (°C)	−50 to +60	80 to 95	90 to 110
Crystallinity	High-crystalline	Low-crystalline	Semi-crystalline
Electrical insulation	Excellent	Excellent	Excellent
Water absorption	Very low	Very low	Low
Recycling Code	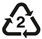	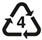	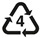
Characteristics	Higher tensile strength	High impact strength	Higher tensile and impact strength
Application	Low-molded bottles for milk, grocery bags, construction film, agricultural mulch, injection-molded pails, caps, appliance housings, and toys	Packaging film, garbage and grocery bags, agricultural mulch, wire, and cable insulation, squeeze bottles, toys, and housewares	Liquid containers, paperboard packaging, stronger films, etc.

**Table 4:** Properties of aromatic polyester compound [71].

Properties	Phthalic Anhydride	Isophthalic Acid	Terephthalic Acid	2,6-Naphthalene Dicarboxylic Acid
Melting point (°C)	131	341–343	427	>300
Density (g/cm^3^)	1.53	1.53	1.522	1.5
Boiling point (°C)	295	412.3	Decomposes	437.3
Molecular weight (g/mol)	148.1	166.14	166.13	216.192
Chemical formula	C_8_H_4_O_3_	C_8_H_6_O_4_	C_8_H_6_O_4_	C_12_H_8_O_4_
Solubility in water	Respond slowly	Insoluble	Soluble	Soluble

**Table 5:** Some important types of nylon composite [30].

Properties	Aluminum-Filled Nylon	Glass-Filled Nylon	Carbon Fiber-Filled Nylon
Tensile Strength (MPa)	48 ± 3	51 ± 3	76 ± 34
Density (g/cm^3^)	1.36 ± 0.05	1.22 ± 0.03	1.9 ± 1.2
Heat Deflection Temperature(°C)	130	110	143
Ball Indentation Hardness	-	98	270
Flexural Modulus(MPa)	3600 ± 150	2900 ± 150	10,300 ± 2070
Elongation at Break(%)	3.5 ± 1	6 ± 3	
Tensile Modulus(MPa)	3800 ± 150	3200 ± 200	7600 ± 2300

**Table 6:** Chemical composition of different types of GFs by wt.% [73,76,77,81].

GFs Type	Oxide											
	SiO_2_	Al_2_O_3_	TiO_2_	B_2_O_3_	CaO	MgO	BaO	LiO_2_	Fe_2_O_3_	F_2_	ZrO_2_	Na_2_O + K_2_O
A-glass	63–72	3.5	0–6	1.5	6.5	4.5	---	---	0–6	0–6	---	14–16
E-glass	54–62	14	0.2	7.0	22	1.0	---	---	0–2	0–1	---	0–2
C-glass	65	4.1	---	5.0	13.4	3.3	0–1	---	0–0.8	---	---	7–10
AR-glass	55–75	0–5	0–12	0–8	1–10	---	---	1–18	0–0.8	0–5	1–18	0–5
S-2-glass	65–66	24–25	---	---	0–0.1	9.5–10	---	---	0–0.1	---	---	0–0.2
D-glass	74	---	---	22.5	0–1	---	---	---	0–0.3	---	---	0–4
R-glass	60	24	---	---	9–25	3–8	---	---	---	0–0.3	---	0–1

**Table 7:** Physical characteristics of various types of carbon fiber [37,108,112,113,114,116].

Type of Fiber	Precursor Material	Density (kg/m^3^)	Tensile Strength [GPa]	Tenacity (GPa)	Young’s Modulus (GPa)	Maximum Elongation (%)
HTS	PAN	1760	3–5	2.8–4	200–250	1.2–1.4
UHTS	PAN	1820	---	4.1–5.7	260–290	0.8–1.0
LM	Pitch	1500	2–4	0.6–1.0	200–250	2.0–5.0
IM	Pitch	1780	4–7	---	250–350	1–2
HM	PAN/Mesophase pitch	1820	2–4.5	1.7–3.5	350–450	0.6–0.7
UHM	Mesophase pitch	2100	3	2.1–2.4	520–550	0.3–0.4

**Table 8:** Main properties of boron fiber [128].

Properties	Unit	Value
Tensile strength	(GPa)	3–4
Density	(g/cm^3^)	2.48–2.83
Young’s modulus	(GPa)	380–400
Melting temperature	(°C)	2040
Coefficient of thermal expansion	(°C^−1^)	8.3 × 10^−6^
Elastic modulus	(GPa)	379.21
Tensile elongation	(%)	0.9
Compression Strength	(GPa)	>6
Hardness	(Knoop)	3200

**Table 9:** Properties of silicon carbide fiber [142].

Properties	Unit	Value
Density	(g/cm^3^)	3.02
Bending strength	(MPa)	250 (20 °C)
	(MPa)	280 (1200 °C)
Elastic modulus	(GPa)	330 (20 °C)
	(GPa)	300 (1200 °C)
Thermal conductivity	(W/mk)	45 (1200 °C)
Specific heat	(J/Kg °K)	750
Thermal expansion coefficient	(K^−1^ × 10^6^)	4.5
Mohs hardness	(−)	13
Acid and alkali resistance	(−)	Excellent
Flexural strength	(MPa)	550
Compressive strength	(MPa)	3900
Volume resistivity	(Ohm cm)	10^2^–10^6^
Maximum service temperature	(°C)	1650

## Data Availability

The data that support the findings of this study are available from the corresponding author, [Emanoil Linul], upon reasonable request.
